# Changes in health-related lifestyle choices of university students before and during the COVID-19 pandemic: Associations between food choices, physical activity and health

**DOI:** 10.1371/journal.pone.0286345

**Published:** 2023-06-23

**Authors:** Greta Jakobsdottir, Runa Sif Stefansdottir, Sunna Gestsdottir, Vignir Stefansson, Erlingur Johannsson, Vaka Rognvaldsdottir, Thordis Lilja Gisladottir

**Affiliations:** 1 Center of Sport and Health Sciences, School of Education, University of Iceland, Reykjavik, Iceland; 2 Department of Sport, Food and Natural Sciences, Western Norway University of Applied Sciences, Bergen, Norway; Al-Ahliyya Amman University, JORDAN

## Abstract

The COVID-19 pandemic has had a profound effect on our lives and society, influencing both individuals’ lifestyles and habits. Recent research shows that anxiety and loneliness have continued to rise, along with changes in food and lifestyle choices. The aim of the study was to investigate whether the pandemic influenced food choices and consumption of energy drinks, alcohol, fruits, and vegetables among first-year university students. Additionally, assessing the relationship between mental and physical health, physical activity, and food choices. A total of 128 male and 128 female first-year students at the University of Iceland were invited to answer an electronic questionnaire in January and early February 2021. A total of 118 students (54% men) participated in the study and valid answers were 115 (46% participation rate). Almost half of the students (44%) experienced that their food choices had worsened, while 14% reported an improvement, compared to before the pandemic. Consumption of caffeinated beverages increased for 26% of students, while 19% experienced a decrease. Just over half of the students reported not drinking alcohol (13%) or reduced consumption (41%). Participants who reported that their mental health had deteriorated or remained the same tended to spend less time on physical activity and experienced worsened food choices (p<0.05). Similarly, those who spent less or the same time on physical activity estimated that their food choices had deteriorated (p<0.05). The COVID-19 pandemic has had a significant impact on the lifestyles of university students and this study has revealed how lifestyle choices and mental health seem to be highly affected by one another. Additionally, the potentially harmful effects of excessive intake of energy drinks need to be enhanced. Interestingly, about 40% of the respondents in the current study drank less alcohol during the pandemic than prior to the pandemic, indicating a strong relationship between alcohol drinking and social gatherings. This study reveals the importance of educating young people on healthy lifestyle choices and the importance of mental health needs to be emphasised.

## Introduction

The COVID-19 pandemic had a major impact on people’s lives. Mental health of Icelandic youth had deteriorated for years before the pandemic hit [1] and the number of individuals experiencing anxiety, depression, and distress continued increase during the pandemic [2–5]. Additionally, loneliness increased during the pandemic [5] and considerable changes were seen in food choices and consumption patterns [6]. It is important to keep in mind that changes in food choices during the COVID-19 pandemic were multifaceted. The number of home-cooked meals increased, which could indicate a healthier consumption pattern, however, greater consumption of sweets and snacks could also be seen, and therefore resulting in a less healthy choices [7]. Various factors can influence changes in food choices in this context, including changes in financial capability, food availability, or university students relocating home to parents or other relatives [8]. Sudden changes in disposable income can lead to short-term food insecurity and studies have shown that individuals living with any level of food insecurity often rely on cheaper and less nutritious foods that often are high in sugar and fat [9,10]. Boredom, anxiety, stress, and lack of daily structure can also lead to changes in eating habits for some, resulting in excessive consumption, especially snacking in-between meals [11–13].

There is little information about food choices and possible changes in relation to consumption of Icelanders during the pandemic. Recent results from a national diet survey showed that around one in five participants believed that the pandemic had affected their diet in a positive or negative way [14]. Improved food choices mainly means a diet with greater consumption of fruits and vegetables, more whole grain and dietary fibre, and less consumption of sugar-sweetened beverages, processed meat [15] and sweets [16,17]. While the opposite would indicate worse food choices. A study conducted during the pandemic found a correlation between hours spent in on-line learning and the consumption of sugary beverages and skipping breakfast [18]. Interestingly, another study showed that feelings of anxiety and other negative emotions have been linked to higher consumption of high-caloric foods, that are often high in added sugar and fat [19]. A review article conducted during the pandemic found similar results showing increased snacking, number of meals, and frequency, but also more cooking from scratch and using fresh produce, and less time spent on physical activity [20].

It is widely known the physical activity has many positive effects on general health, both physical and mental [21]. Physical antivity can aid in depression and anxiety [22], as well improve sleep quality [23]. Physical activity has also been shown to enhance brain and cognitive functioning [24,25]. Prolonged sedentary behavior and inactivity has been associated with many negative health outcomes like cardiovascular disease, type 2 diabetes, some types of cancer and all-cause mortality [26–28]. Therefore, keeping an active lifestyle is of great importance. The pandemic affected both level and intensity of an individual’s physical activity. When looking into time spent on physical activity of university students, most studies show a reduced time spent on physical activity and increased sedentary time [29–31], negativelly influencing the students’ well-being, strength, endurance, and BMI [31].

Starting some years before the pandemic, the consumption of energy drinks among teenagers and young adults in Iceland has increased dramatically, and the most in recent years. In 2020, about half of students in secondary schools consumed at least one serving of energy drink per day [32]. Increased consumption of energy drinks among young people can also be seen in neighboring countries [33]. Energy drinks contain mixtures of various substances, for example, amino acids, sweeteners, and vitamins, while all contain a high amount of caffeine. Most energy drinks contain approximately 80–350 mg of caffeine per serving. In comparison a standard cup of coffee contains about 100 mg of caffeine [34]. Long-term excessive consumption of energy drinks, coffee, and related products can have harmful effects on health. The main negative effects of high caffeine consumption are sleep disorders and disturbances in sleep quality [35–37]. High consumption of energy drinks is also linked to tooth enamel erosion [38], while physical complaints include headaches, nausea, stomachaches, and diarrhea [39], increased blood pressure, and a faster heart rate [39–41]. Additionally, the consumption of energy drinks, especially when mixed with alcohol, can promote anxiety and irritability [37,42–44]. Caffeine consumption has a particularly harmful effect on children and young individuals and can be one of the contributing factors to the reported short sleep among this age groups [45,46], especially when caffeine is consumed shortly before bedtime [47].

The results of recent studies indicate that alcohol consumption during COVID-19 increased, especially among those who experienced stress or anxiety [48,49]. The alcohol consumption of many Icelanders during the first few months of the pandemic did not change, according to the Directory of Health, while a third estimated that they consumed less or 15% more [50]. Similar results can be found elsewhere [49].

The aim of this study was to examine self-reported food choices, alcohol, and caffeine intake among first-year Icelandic university students during COVID-19 compared to before the pandemic. Additionally, the association between mental and physical health, physical activity, and food choices was examined.

## Methods

The participants in the current study were young adult university students, selected from enrolled first-year students at the University of Iceland. A total of 495 students, 128 males and 366 females, fit the criteria and all 128 male students and 128 randomly selected female students were offered participation. The age-limit of 32 years was set for general comparison to the age group young adults, which was the focus group of the study. Students were sent a questionnaire through their student email in January and early February of 2021 resulting in 118 participants, 63 males and 54 females. Three participants were excluded due to invalid or implausible answers. Further information regarding participants and data collection is described in detail in a prior publication [5]. Participants provided written consent, and study procedures were conducted according to the guidance provided in the Declaration of Helsinki. The National Bioethics Committee and the Icelandic Data Protection Authority approved the study (VSNb2020100026/03.01).

### Measures

#### Food and drinks

Food and beverage consumption was estimated using standardized questions from the Icelandic Center for Social Research and Analysis (ICSRA). Perceived food consumption was estimated with seven questions; “*How often do you eat/drink fruit and berries/vegetables/energy drinks*”. There were 8 possible responses ranging from 1 (never) up to 8 (three times a day or more). Participants were also asked to estimate changes in their personal food choices and compare general food consumption before and during the pandemic with the following question: “*How do you estimate your food choices compared to the time before COVID-19*?”. Response options were worse, similar, and improved.

#### Alcohol consumption

Alcohol consumption was estimated with the following question: “*How do you estimate your alcohol consumption compared to the time before COVID-19*?”. Response options were: I do not drink alcohol, I drink less, I drink a similar amount, and I drink more.

#### Caffeine consumption

Caffeine consumption was assessed with the three following questions. “*How do you estimate the amount consumed of caffeinated beverages compared to the time before COVID-19*?”. Response options were: I do not drink caffeinated beverages, I drink less, I drink a similar amount, and I drink more. “*What is the average amount of caffeine in your preferred energy drink*?” with the following response options: approximately (appr.) 80 mg, appr. 105 mg, appr. 180 mg, and I do not know. The final question was regarding the time of consumption: “*When during the day do you consume energy drinks*”. Response options were given in the following time intervals: before 1 pm, between 1 and 5 pm, and after 5 pm.

#### Mental and physical health

The status of mental and physical health was estimated with the following questions. “*How do you estimate your physical health compared to the time before COVID-19*” and “*How do you estimate your mental health compared to the time before COVID-19*”. Response options were worse, the same, and better. Regarding physical activity the following question was included: “*During COVID-19*, *how do you estimate your physical activity compared to the time before COVID-19*”, and the response options were: less, equal, and more.

#### Demographics

Participants reported their age (birth year), gender, height (cm), and weight (kg). Body Mass Index (BMI) (kg/m^2^) was calculated with the appropriate measures. Participants were asked if they had been digonsed with COVID-19, and response options were yes, no, and I do not want to answer.

### Statistical analyses

Descriptive summaries are presented as means and standard deviations for continuous variables and as frequencies and percentages for categorical variables. In addition, study variables were analyzed for distributional properties. The alpha level for significant differences was set at 0.05. To assess gender differences, an independent-sample t-test was used for continuous variables and a chi-square test for categorical variables. Statistical analyses were performed in Jamovi, edition 2.2.

## Results

### Background information

The average age of the students was 24.1 years, the average height and weight of male students were 183.2 cm and 80 kg, respectively, while the average height and weight of females was 167.3 cm and 70.9 kg, respectively. The average BMI of males and females was 23.9 kg/m^2^ and 24.7 kg/m^2^, respectively (Table 1). Only two male participants (1.7%) had been tested positive for COVID-19, and therefore having minimal impact on the results. Results from a prior publication showed no effect of social economic status on mental health or physical activity [5].

**Table 1:** Background information on participants.

	Number	All	Male	Female	p-value
		(SD)	(SD)	(SD)	
**Number (%)**		115 (100)	62 (54)	53 (46)	
**Age (year)**	115	24.1 (3.4)	24.1 (3.1)	24.1 (3.7)	0.933
**Height (cm)**	115	176.0 (10.2)	183.2 (6.7)	167.3 (6.0)	< 0.001
**Weight (kg)**	109	75.8 (13.4)	80.0 (11.3)	70.9 (14.1)	< 0.001
**BMI (kg/m** ^**2**^ **)**	110	24.2 (4.5)	23.9 (3.3)	24.7 (5.7)	0.358
**Positive COVID-19 test**	115	2 (1.7%)	2 (3.2%)	0	0.190
**Icelandic as a native language**	115	111 (96.5%)	60 (96.8%)	51 (96.2%)	0.642

### Food consumption

Nearly half of the students (44%) estimated that their food consumption had worsened compared to before the pandemic and only 14% rated their food choices as better (Table 2). It is important to keep in mind that the questions asked the participants to subjectively estimate changes in their own diet and food choices. Of the students who responded that their diet had worsened, 31% ate fruits and berries, and 43% vegetables at least once to three times a day or more. Considering those who rated that their food choices had improved from before the pandemic, 44% of the participants ate fruits and berries, while 56% ate vegetables at least one to three times a day or more. A total of 15 respondents ate two to three servings of both fruits and vegetables per day (Table 3). No difference could be seen regarding gender differences on changes in food consumption.

**Table 2:** Information on how participants experienced changes in their food choices, consumption of energy drinks and alcohol prior compared to during COVID-19.

					Gender		
	Response	All			Male	Female	p–value*
**Food choices**	Worse	51		44.3%	32 (51.6%)	19 (35.8%)	0.228
	Similar	48		41.7%	22 (35.5%)	26 (49.1%)	
** **	Improved	16		13.9%	8 (12.9%)	8 (15.1%)	
**Alcohol consumption**	I do not consume alcohol	15		13.0%	7 (11.3%)	8 (15.1%)	0.183
	I drink less	47		40.9%	31 (50.0%)	16 (30.2%)	
	I drink similar amounts	37		32.2%	16 (25.8%)	21 (39.6%)	
** **	I drink more	16		13.9%	8 (12.9%)	8 (15.1%)	
**Energy drink consumption**	I do not consume energy drinks	13		11.3%	7 (11.3%)	6 (11.3%)	0.862
	I drink less	22		19.1%	13 (21.0%)	9 (17.0%)	
	I drink similar amounts	50		43.5%	27 (43.5%)	23 (43.4%)	
	I drink more	30		22.6%	15 (24.2%)	15 (28.3%)	

*Gender comparison.

**Table 3:** How does consumption of fruits, berries and vegetables, alcohol and caffeine reflected in how individuals evaluate their food choices?.

During COVID-19, how do you evaluate your food choices compared to prior to the pandemic?	Worse		Similar		Better		Total	
	Number (n)	Ratio (%)	Number (n)	Ratio (%)	Number (n)	Ratio (%)	Number (n)	Ratio (%)
Total	51	44.3%	48	41.7%	16	13.9%	115	100%
**How many times a week do you consume fruits and berries?** ^**a**^								
Seldomly	8	16%	5	10%	2	13%	**15**	**13%**
Sometimes	27	53%	24	50%	7	44%	**58**	**50%**
Often	16	31%	19	40%	7	44%	**42**	**37%**
**How many times a week do you consume vegetables?** ^**a**^								
Seldomly	7	14%	4	8%	0	0%	**11**	**10%**
Sometimes	22	43%	31	65%	7	44%	**60**	**52%**
Often	22	43%	13	27%	9	56%	**44**	**38%**
**During COVID-19, how do you evaluate your alcohol consumption compared to before the pandemic**								** **
I do not drink alcohol	8	16%	5	10%	2	13%	**15**	**13%**
I drink less alcohol	18	35%	21	44%	8	50%	**47**	**41%**
I drink similar amount of alcohol	17	33%	15	31%	5	31%	**37**	**32%**
I drink more alcohol	8	16%	7	15%	1	6%	**16**	**14%**
**During COVID-19, how do you evaluate your consumption of caffeinated beverages compared to before the pandemic**								** **
I do not drink caffeinated beverages	4	8%	9	19%	0	0%	**13**	**11%**
I drink fewer caffeinated beverages	5	10%	11	23%	6	38%	**22**	**19%**
I drink similar amounts of caffeinated beverages	25	49%	18	38%	7	44%	**50**	**43%**
I drink more caffeinated beverages	17	33%	10	21%	3	19%	**30**	**26%**

^a^ Seldomly: Never, less then 1x per week, once a week; Sometimes: 2-3x per week and 4-6x per week; Often: Once a day, 2x per day and 3x a day or more.

### Caffeine consumption, timing, and quantity

A large proportion of students, 44%, considered their consumption of caffeinated beverages to be similar to before the beginning of the pandemic, but about 19% considered their caffeine consumption to be less, while a quarter considered it greater (Table 2). No difference could be seen regarding gender differences in changes in energy drinks or alcohol consumption.

Of those who rated that their food choices had worsened, only 10% drank less caffeine compared to 38% of those who rated that their food choices had improved. If looking at those who thought they drank more caffeine, 33% rated their food choice as worse compared to 19% of those who rated it better (Table 3).

A large number of the participants consumed their caffeinated beverages before 5 pm, with the majority of students consuming them before 1 pm, 40% consuming energy drinks between 1 pm and 5 pm, and only 12% consumed one or more after 5 pm. Most of the students, or about 90%, consumed two or fewer energy drinks during the day and about 70% consumed one or no energy drinks during the day. Male students consumed more energy drinks, compared to females, with almost 40% of them consuming two to five servings, compared to only 20% of female students consuming two to three servings. No female reported drinking more than three drinks per day. The most common amount of caffeine in the drinks was 105 mg, which is similar to the amount found in one serving of a regular cup of coffee. The estimated total amount of caffeine from energy drinks was approximately 105–525 mg (1–5 servings) for males compared to 105–315 mg (1–3 servings) for females. When looking at the consumption of caffeinated beverages (coffee and energy drinks that contain caffeine) during the week, about 23% of respondents rarely or never consume caffeinated beverages, 36% sometimes (2–6 times a week) and 53% consume caffeinated beverages once a day or more (S1 Table).

### Alcohol consumption

One in three students consumed a similar amount of alcohol during COVID-19 compared to before the pandemic, but more than half did not consume alcohol or consumed less (13% and 41%, respectively). On the other hand, 14% of students consumed more alcohol at the time of COVID-19 than before the pandemic (Table 2). Of the students who rated their food choices worse, about half of the students consumed the same or more alcohol (33% and 16% respectively). Conversely, among those who rated their food choices better, half drank less alcohol, compared to 35% of those who rated their food choices worse (Table 3).

### The relationship between mental and physical health, physical activity and food choices

**Fig 1:**
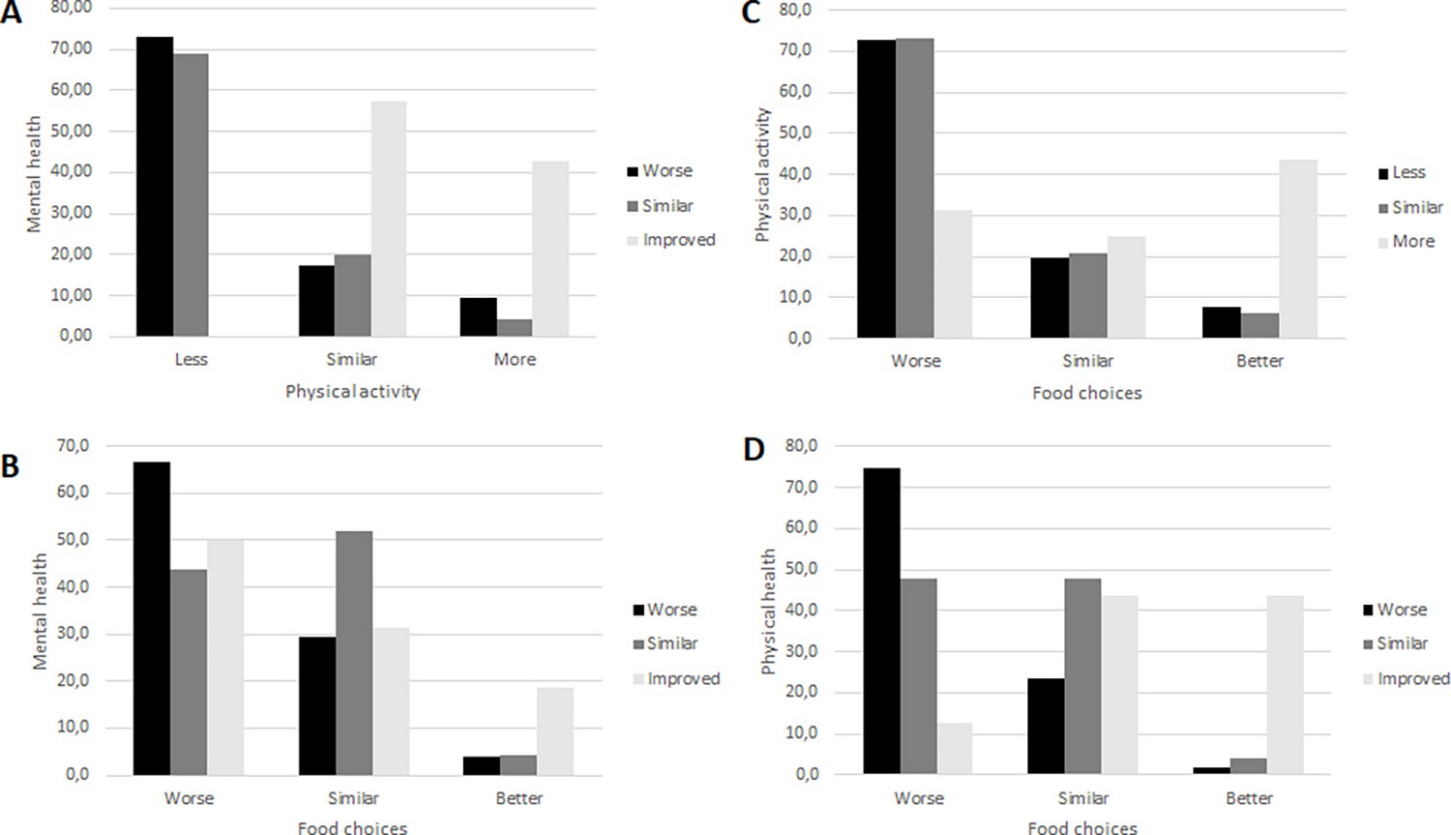
The relationship between mental and physical health (1A), mental health and food choices (1B), physical activity and food choices (1C); and physical health and food choices (1D).

The participants that reported that their mental health had deteriorated or remained similar tended to spend less time on physical activity (73% and 69% respectively) (Fig 1A). However, those who reported improved mental health exercised either for the same amount of time (57%) or more (43%) (p<0.05). Additionally, none of those who spent less time on physical activity experienced improvement in their mental health.

A similar pattern could be seen when looking into mental health and food choices. Participants that reported worsened food choices were more likely to have experienced deterioration in their mental health (p<0.05) (Fig 1B). Two-thirds of the participants reported that their mental health had worsened and that their food choices had deteriorated as well (67%), compared to similar food choices (29%) or better food choices (4%). Those who rate their mental health as improved were the group most likely to rate their food choices as better (19%) (Fig 1B).

A large number of participants (73%) reported spending less or similar time on physical activity, and that their food choices had deteriorated, while one-third that spent more time on physical activity still had worsened food choices (Fig 1C). However, 44% of those who reported spending more time physically active stated that their food choices had improved (p<0.001). Similarly, when looking at physical health and food choices, worse physical health was negatively associated with improved food choices, such that worse physical health was associated with poorer food choices and the other way around (p<0.001) (Fig 1D). More male students students estimated that their physical health had worsended compared to female students during the pandemic (p<0.002) (S2 Table).

## Discussion

The main findings of this study reveal a clear correlation between mental and physical health and food choices of university students during the COVID-19 pandemic indicating that worsening health is associated with unhealthier food choices. Additionally, when compared to the time before the pandemic, more than half of the participants considered that their food choices had become less healthy (deteriorated), one in five students drank fewer energy drinks and around 40% of students consumed less alcohol during the pandemic.

The pandemic has had a major impact on the lives and well-being of university students. University students are often faced with major life changes and challenges, for example in their studies, but also due to economic and social responsibilities [51] and can therefore be a vulnerable social group, even before the pandemic started. A similar number of students lived on their own and with parents or other relatives, however, information regarding changes in living arrangements is missing. It can be assumed that many students moved to their parents or other relatives when the pandemic prolonged.

Social restrictions due to the pandemic had a considerable impact on the learning environment of university students. Teaching, class time, and group work primarily moved to an electronic format with distance learning. Students, therefore, did not attend school and classes in the traditional way due to access to classes and teachers being online, presumably at home. According to students, working on group assignments became more difficult and loneliness did increase. However, the quality of distance learning and technical aspects did improve [52]. This change affected social relationships and interactions between students and also their teachers, as social restrictions meant that there was little to no interaction between students at school and during leisure hours. Social interactions and activities that often involved food and drinking became almost nonexistent.

### Factors influencing food choices

Many different factors can affect students’ eating habits. Studies looking at eating behavior during the pandemic show similar results, that is, students eat both less and more healthfully [7]. More students thought that their diets had become less healthy (deteriorated) during the pandemic compared to those who estimated that their diets had become healthier (improved). Some students may have had to move home to their parents or other relatives during the pandemic, however, information on the change of housing conditions is not available in the current study. One can speculate that, when moving home to relatives, students may not be responsible for grocery shopping and might even have limited access to food or have fewer items to choose from. All these factors can have a major impact on food choices and eating behaviors. According to a Spanish study on university students, most shopped for groceries once or less per week [53].

Other factors that might have influenced food choices during the pandemic includes more time spent in the kitchen, like baking and cooking, and eating more frequently due to boredom, anxiety, and distress over the situation [6,7,53]. More time spent cooking and baking together with a higher frequency of home-cooked meals can lead to both healthier and unhealthier eating habits. Limited access to dine-in restaurants during the pandemic may have also led to more home-cooked meals, resulting in somewhat healthier food [7]. Fast-food restaurants offering takeaway options were on the other hand open for business, which could have increased the number of fast-food meals consumed.

First-year Icelandic university students experienced deterioration in their mental health during the pandemic, as well as increased loneliness, stress, and reduced physical activity. Males experienced greater deterioration in the physical health, while females felt more anxious, depressed and lonely [5]. Participants in the current study estimated that their mental health did deteriorate, and that could be correlated with both less physical activity and negative changes in food choices. The same can be said for both physical health and physical activity in relation to food choices. Mental health has in other studies been linked to less healthy food consumption but what is different in the context of COVID-19 is that external factors had the greatest impact, which was out of the individual’s control.

Similar results have been seen elsewhere [54] and highlight the importance of promoting both physical and mental health. In a Spanish longitudinal study, where the sample was mostly made of university students (18 years and older), survey data from the beginning of the quarantine and again five days after the quarantine was eased showed a decrease in physical activity, sleep quality, mental health and increase in body weight. The researchers speculated that weight gain might be due to decreased physical activity and changes in food consumption [55]. In the current study, however, changes in body weight were not included in the questionnaire. Other studies, on mental health, conducted during the COVID-19 pandemic have found increased anxiety and depression [56,57].

It is noteworthy, that about half of the university students estimated that their food choices had worsened during COVID-19, however, few of those were close to meeting or did meet the official recommendations regarding daily consumption of fruits and vegetables, five servings a day [58]. It is, however, important to keep in mind, that only 16 students estimated that their food choices had improved during the pandemic, compared to 51 who thought it had worsened. Generally, university students did not meet the “5 a day” fruits/vegetables recommendations, and only about 20% eat two or more servings of fruits and vegetables per day.

### Caffeinated beverages

During the pandemic, about 73% of university students consumed 1–2 caffeinated beverages per day. The most common amount of caffeine in these energy drinks is 105 mg, resulting in caffeine consumption within safe limits (400 mg/day) [59]. In total, 27 students (23%) reported regular consumption of both coffee and energy drinks, which can result in a considerably higher intake of caffeine. However, less than 15% of university students drank two or more cups of coffee a day, and about a third of them coffee in addition to dranking energy drinks 4–6 times a week or more. Furthermore, the timing of the consumption of energy drinks is sensible, as the great majority of the students consume them before 5 pm. Recommendation regarding consumption of caffeinated beverages is to consume caffeine early during the day and preferable not after appr. 3 pm, as the half-life of caffeine in the body of healthy, non-smokers is around 4–6 hours [60]. It is quite common that individuals consume caffeine to sharpen and maintain concentration, just as they are marketed for, however, the effects of caffeine on concentration and learning ability are unclear [61].

### Consumption of alcohol

Interestingly, almost half of university students consumed less alcohol during the pandemic compared to before the pandemic. Compared to a national Icelandic survey [50], university students decreased their alcohol consumption to a greater extent than the average adult. In that survey, 56% had unchanged consumption, 29% consumed less, and 15% increased their consumption [50]. A similar percentage of students in the current study increased their consumption, or 14%.

Alcohol consumption in general of university students has not been surveyed in Iceland, however, alcohol consumption by teenagers and young adults has been low in recent years. A large European study from 2019 showed that 37% of Icelandic youth (15–16 years old) had consumed alcoholic beverages at least once in their life [62] and a survey from 2020, showed that only one out of four high school students had become intoxicated once or more in 30 days period [32,63]. As stated earlier, there is a lack of research on the alcohol consumption of university students, but the results of this study show that a large number of university students changed their alcohol consumption for the better and consumed less alcohol compared to the general Icelandic population during the pandemic. Furthermore, 13% of the students do not drink alcohol and live an alcohol-free lifestyle. As a result, social restrictions could have had a positive effect on the alcohol consumption of university students, as most social events such as entertainment, group gatherings, and parties were prohibited, and therefore the social aspect of alcohol consumption seems to be strong when it comes to university students.

### Strength and limitations

The study provides important information and an overview of the food choices, consumption of caffeinated beverages, and alcohol of university students during the COVID-19 pandemic. It is important to consider future research on the long-term effects of the pandemic on young adults, research like the current one gives important information on changes that the participants experienced during these unexpected times. The study is based on a questionnaire where the participants themselves estimated their food choices, consumption of caffeinated beverages and alcohol, as well as physical health and activity, and mental health, which could affect the response and results. The survey did not include visual examples of caffeine amount found in common caffeinated beverages which could make the estimation for some participants inaccurate. However, the amount of caffeine suggested in the questionnaire compares to the amount of the most popular energy drinks that are currently marketed in Iceland. Additionally, caffeine consumption is also impacted by consumption of other beverages than energy drinks and coffee, such as tea and soft drinks. The sample of the study is rather small and homogeneous, although probably typical for university students in Iceland. Finally, the study is a cross-sectional study, and therefore it is difficult to determine causality. For future studies, questions on smoking behavior should be added as smoking can influence alcohol consumption and food choices.

### Conclusions

The COVID-19 pandemic has had a considerable impact on the food choices and consumption habits of university students around the world. However, it is important to realize that the effects are both positive and negative. More than half of the students in this study categorized their food choices as less healthy during the pandemic than before, despite abundant consumption of fruits and vegetables. Food choices seem to be highly impacted by mental and physical health as well as the level of physical activity. One in four students estimated that their consumption of energy drinks was higher, and almost half of students estimated their alcohol consumption was lower than before the pandemic. Young adults’ awareness of the importance of healthy food choices and lifestyle, and especially the potential harm of energy drinks, needs to be further strengthened.

## Supporting information

S1 Table When and how many energy drinks did the participants consume. (DOCX)

S2 Table Information on how participants experienced changes in their physical activity and physical health prior compared to during COVID-19. (DOCX)

S1 File (CSV)
