# Supplementary material for: Changes in health-related lifestyle choices of university students before and during the COVID-19 pandemic: Associations between food choices, physical activity and health
Source: PLoS One. 2023 Jun 23;18(6):e0286345. doi: 10.1371/journal.pone.0286345 (PMC10289399; doi:10.1371/journal.pone.0286345)
Supplement: S1 Table — (DOCX) [file pone.0286345.s001.docx]

S1 table. When and how many energy drinks did the participants consume.

|  | **Total** | | **Female** | | **Male** | |
| --- | --- | --- | --- | --- | --- | --- |
|  | **(n=101)** | | **(n=46)** | | **(n=55)** | |
|  | **n** | **Ratio (%)** | **n** | **Ratio (%)** | **n** | **Ratio (%)** |
| **When do you consumer energy drinks?** | |  |  |  |  |  |
| **Before 1 pm** |  |  |  |  |  |  |
| None | 45 | 44.6% | 23 | 50.0% | 22 | 40.0% |
| One serving | 50 | 49.5% | 23 | 50.0% | 27 | 49.1% |
| 2-3 servings | 6 | 6.0% | 0 | 0% | 6 | 10.9% |
| **Between 1 and 5 pm** |  |  |  |  |  |  |
| None | 58 | 57.4% | 27 | 58.7% | 31 | 56.4% |
| One serving | 41 | 40.6% | 29 | 63.0% | 22 | 40.0% |
| 2-3 servings | 2 | 2.0% | 0 | 0% | 2 | 3.6% |
| **After 5 pm** |  |  |  |  |  |  |
| None | 89 | 88.1% | 43 | 93.5% | 46 | 83.6% |
| One serving | 9 | 8.9% | 2 | 4.3% | 7 | 12.7% |
| 2-3 servings | 3 | 3.0% | 1 | 2.2% | 2 | 3.6% |
| **Total of serving per day** |  |  |  |  |  |  |
| None | 19 | 18.8% | 10 | 21.7% | 9 | 16.4% |
| One serving | 52 | 51.5% | 27 | 58.7% | 25 | 45.5% |
| 2-5 servings | 30 | 27.7% | 9 | 19.6% | 21 | 38.1% |
| **Most common amount of caffeine in the energy drinks you consume?** | | | | | | |
| Appr. 80 mg | 7 | 6.9% | 4 | 8.9% | 3 | 5.5% |
| Appr. 105 mg | 59 | 58.4% | 22 | 48.9% | 36 | 65.5% |
| Appr. 180 mg | 10 | 9.9% | 5 | 11.1% | 5 | 9.1% |
| Do not know | 25 | 24.8% | 14 | 31.1% | 11 | 20.0% |
