# Supplementary material for: Changes in health-related lifestyle choices of university students before and during the COVID-19 pandemic: Associations between food choices, physical activity and health
Source: PLoS One. 2023 Jun 23;18(6):e0286345. doi: 10.1371/journal.pone.0286345 (PMC10289399; doi:10.1371/journal.pone.0286345)
Supplement: S2 Table — (DOCX) [file pone.0286345.s002.docx]

| S2 table. Information on how participants experienced changes in their physical activity and physical health prior compared to during COVID-19. | | | | | | |  |  |  |
| --- | --- | --- | --- | --- | --- | --- | --- | --- | --- |
|  |  |  |  | **Gender** | |  | |  |  |
|  |  | **All** | | **Male** | **Female** | **P – value*** | |  |  |
| Physical activity | Less | 77 | 67.0% | 47 (75.8%) | 30 (56.6%) | 0.088 | |  |  |
|  | Similar | 24 | 20.9% | 9 (14.5%) | 15 (28.3%) |  |  |  |  |
|  | More | 14 | 12.2% | 6 (9.7%) | 8 (15.1%) |  |  |  |  |
| Physical health | Worse | 63 | 54.8% | 43 (69.4%) | 20 (37.7%) | 0.002 | |  |  |
|  | Similar | 42 | 36.5% | 14 (22.6%) | 28 (52.8%) |  |  |  |  |
|  | Improved | 10 | 8.7% | 5 (8.1%) | 5 (9.4%) |  |  |  |  |
| *Gender comparison. | | | | | | | | | |
